# Supplementary material for: Reconstructing Genomes of Carbon Monoxide Oxidisers in Volcanic Deposits Including Members of the Class Ktedonobacteria
Source: Microorganisms. 2020 Nov 27;8(12):1880. doi: 10.3390/microorganisms8121880 (PMC7761526; doi:10.3390/microorganisms8121880)
Supplement: Supplementary file 1 [file microorganisms-08-01880-s001.pdf]

Supplementary Information

Reconstructing genomes of carbon monoxide oxidisers in volcanic deposits including members of the class Ktedonobacteria

Marcela Hernández [1,2,6,*], Blanca Vera-Gargallo [3], Marcela Calabi-Floody [4], Gary M King [5], Ralf Conrad [6] and Christoph C. Tebbe [1]

[1] Thünen Institut für Biodiversität, Braunschweig, Germany, christoph.tebbe@thuenen.de;
[2] School of Environmental Sciences, Norwich Research Park, University of East Anglia, Norwich, UK, marcela.hernandez@uea.ac.uk;
[3] Department of Microbiology and Parasitology, Faculty of Pharmacy, University of Seville, Seville, Spain, vera@us.es;
[4] BIOREN-UFRO, Universidad de La Frontera, Temuco, Chile; marcela.calabi@ufrontera.cl;
[5] Department of Biological Sciences, Louisiana State University, Baton Rouge, LA, USA; gkingme@gmail.com;
[6] Max Planck Institute for Terrestrial Microbiology, Marburg, Germany, conrad@mpi-marburg.mpg.de;

*Correspondence: marcela.hernandez@uea.ac.uk (M.H.)

Supplementary figure legend:
Figure S1. Relative abundance of main phyla based on classification of scaffolds >500 bp. Other phyla include members of Planctomycetes, Gemmatimonadetes, Cyanobacteria, Firmicutes, Armatimonadetes and Patescibacteria.

Supplementary table legends:
Table S1. List of strains used for the tree construction.
Table S2. Summary table of other complete cellular functions in the MAGs isolates form sites 1640, 1751 and 1957 retrieved from KEGG analysis. Reference genomes for Ketedonobacteria: DSM45816T [1], DSM44963^T [2] and NBRC 113551^T [3], (K: KEGG orthology; M: KEGG Mode). Asterisks indicate the MAGs isolated from the class Ktedonobacteria.
Table S3. Summary of the metagenome-assembled genomes (MAGs) isolated in the present study. Average amino-acid identity (AAI) was calculated by comparing the MAGs with their closest reference genomes identified based on the RAST results. The RefSeq accession number of the reference genomes are provided in parenthesis.
Table S4. Summary of enzymatic functions for CO-, H2-, and formate-oxidation in the Ktedonobacteria reference genomes. Carbon monoxide oxidation: K03518 carbon monoxide dehydrogenase small subunit coxS, K03519 carbon monoxide dehydrogenase medium subunit coxM, K03520 carbon monoxide dehydrogenase large subunit coxL. Formate oxidation: K00122 formate dehydrogenase, K00123 formate dehydrogenase major subunit. Hydrogen oxidation: H_2 dehydrogenase: K00436: NAD-reducing hydrogenase large subunit.

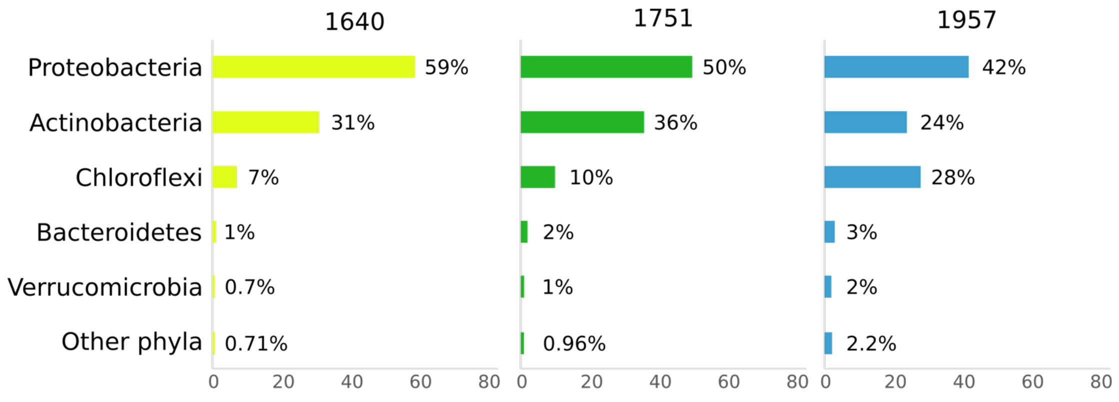

Figure S1. Relative abundance of main phyla based on classification of scaffolds >500 bp. Other phyla include members of Planctomycetes, Gemmatimonadetes, Cyanobacteria, Firmicutes, Armatimonadetes and Patescibacteria.

Table S1. List of strains used for the tree construction.

Strain name	Type strain	NCBI Accession number
Acetobacter aceti TMW2.1153		NZ_CP014692.1
Acetobacter orleanensis JCM 7639	yes	NZ_BAMY01000269.1
Acetobacter pasteurianus IFO 3283-01		NC_013209.1
Acetobacter syzygii 9H-2		NZ_BAMZ01000094.1
Acidipila dinghuensis DHOF10	yes	NZ_SDMK01000001.1
Acidipila rosea DSM 103428		NZ_SMGK01000001.1
Acidisphaera rubrifaciens HS-AP3	yes	NZ_BANB01001239.1
Acidobacterium capsulatum ATCC 51196	yes	NC_012483.1
Acidocella aminolytica 101	yes	NZ_FQVJ01000109.1
Actinosynnema mirum DSM 43827	yes	NC_013093.1
Ammonifex degensii KC4	yes	NC_013385.1
Asaia bogorensis NBRC 16594	yes	NZ_AP014690.1
Bacteroides fragilis YCH46		NC_006347.1
Bacteroides ovatus ATCC 8483	yes	NZ_CP012938.1
Bacteroides uniformis ATCC 8492	yes	NZ_DS362249.1
Bacteroides vulgatus ATCC 8482	yes	NC_009614.1
Bacteroides dorei CL03T12C01		NZ_CP011531.1
Beutenbergia cavernae DSM 12333	yes	NC_012669.1
Bryobacter aggregatus MPL3	yes	NZ_JNIF01000001.1
Candidatus Koribacter versatilis Ellin345		NC_008009.1
Candidatus Solibacter usitatus Ellin6076		NC_008536.1
Chloroflexus aurantiacus J-10-fl	yes	NC_010175.1
Dictyobacter aurantiacus S-27	yes	NZ_BIFQ01000001.1
Dictyobacter alpinus Uno16	yes	NZ_BIFT01000001.1
Dictyobacter kobayashii Uno11	yes	NZ_BIFS01000001.1
Dictyobacter vulcani W12	yes	NZ_BKZW01000001.1
Edaphobacter aggregans DSM 19364	yes	NZ_JQKI01000001.1
Edaphobacter modestus DSM 18101	yes	NZ_SHKW01000001.1
Gemmatimonadetes bacterium 2013_60CM_65_52		NGFI01000005.1
Gemmatimonas aurantiaca T-27	yes	NC_012489.1
Gemmatimonas phototrophica AP64	yes	NZ_CP011454.1
Gemmatirosa kalamazoonesis KBS708	yes	NZ_CP007128.1
Gluconacetobacter diazotrophicus PAl 5	yes	NC_011365.1
Gluconacetobacter entanii LTH 4560	yes	NZ_NKUF01000001.1
Gluconobacter kanchanaburiensis NBRC 103587	yes	NZ_BJVA01000001.1
Gluconobacter oxydans H24		NC_019396.1
Gluconobacter roseus LMG 1418		NZ_LHZP01000023.1
Gluconobacter sphaericus NBRC 12467	yes	NZ_BJMK01000001.1
Gluconobacter thailandicus F149-1	yes	NZ_BANH01000191.1
Gluconobacter wancherniae NBRC 103581	yes	NZ_BJUZ01000001.1
Granulicella mallensis MP5ACTX8	yes	NC_016631.1
Granulicella tundricola MP5ACTX9	yes	NC_015064.1
Herpetosiphon aurantiacus DSM 785	yes	CP000875.1

continuation...

Strain name	Type strain	NCBI Accession number
Komagataeibacter europaeus SRCM101446		NZ_CP021467.1
Komagataeibacter hansenii ATCC 23769		NZ_CM000920.1
Komagataeibacter oboediens 174Bp2		NZ_CADT01000200.1
Komagataeibacter xylinus E25		NZ_CP004360.1
Ktedonobacter racemifer DSM 44963	yes	NZ_ADVG01000010.1
Ktedonosporobacter rubrisoli SCAWS-G2	yes	NZ_CP035758.1
Mycobacterium tuberculosis H37Rv	yes	NC_000962.3
Mycobacterium vanbaalenii PYR-1	yes	NC_008726.1
Nocardia farcinica NCTC11134	yes	NZ_LN868938.1
Nocardiopsis dassonvillei DSM 43111	yes	NC_014210.1
Prosthecobacter fusiformis ATCC 25309	yes	NZ_SOCA01000001.1
Prosthecobacter debontii ATCC 700200	yes	NZ_FUYE01000055.1
Rhodococcus jostii RHA1		NC_008268.1
Roseiflexus castenholzii DSM 13941	yes	NC_009767.1
Roseimicrobium gellanilyticum DSM 25532	yes	NZ_QNRR01000001.1
Roseomonas gilardii U14-5		NZ_CP015583.1
Saccharomonospora viridis DSM 43017	yes	NC_013159.1
Saccharopolyspora erythraea NRRL2338	yes	NC_009142.1
Salinispora arenicola CNS-205		NC_009953.1
Salinispora tropica CNB-440	yes	NC_009380.1
Sphaerobacter thermophilus DSM 20745	yes	NC_013523.1
Streptomyces avermitilis MA-4680	yes	NC_003155.5
Streptomyces coelicolor A3(2)		NC_003888.3
Streptomyces griseus subsp. griseus NBRC 13350		NC_010572.1
Streptomyces scabiei 87.22		NC_013929.1
Streptosporangium roseum DSM 43021	yes	NC_013595.1
Tengunoibacter tsumagoiensis Uno3	yes	NZ_BIFR01000001.1
Terriglobus albidus ORNL		NZ_CP042806.1
Terriglobus roseus DSM 18391	yes	NC_018014.1
Terriglobus saanensis SP1PR4	yes	NC_014963.1
Terriglobus sp. TAA 43		NZ_JUGR01000001.1
Thermobifida fusca YX		NC_007333.1
Thermogemmatispora carboxidivorans PM5	yes	NZ_JNIM01000001.1
Thermogemmatispora tikiterensis T81	yes	NZ_MCIF01000002.1
Thermogemmatispora aurantia A1-2	yes	NZ_BKZV01000001.1
Thermogemmatispora onikobensis NBRC 111776	yes	NZ_BDGT01000001.1
Thermomicrobium roseum DSM 5159	yes	NC_011959.1
Thermomonospora curvata DSM 43183	yes	NC_013510.1
Thermosporothrix hazakensis SK20-1	yes	NZ_BIFX01000001.1
Verrucomicrobium spinosum DSM 4136	yes	NZ_ABIZ01000001.1
Verrucomicrobium sp. GAS474		LT629781

Table S2. Summary table of other complete cellular functions in the MAGs isolates form sites 1640, 1751 and 1957 retrieved from KEGG analysis. Reference genomes for Ketedonobacteria: DSM45816T [1], DSM44963^{T} [2] and NBRC 113551^{T} [3], (K: KEGG orthology; M: KEGG Mode). Asterisks indicate the MAGs isolated from the class Ktedonobacteria.

	1640		1751	1957																Reference		
**Function (M: KEGG Mode)**	MAG-1.1	MAG-2.1	MAG-1.1	MAG-1.1	MAG-2.1 *	MAG-3.1 *	MAG-4.1	MAG-5.1	MAG-6.1 *	MAG-7.1	MAG-8.1	MAG-9.1	MAG-10.1	MAG-11.1	MAG-12.1	MAG-13.1	MAG-14.1	MAG-15.1	MAG-16.1	DSM45816^{T}	DSM44963^{T}	NBRC113551^{T}
**Fatty acid metabolism**																						
M00082 Fatty acid biosynthesis, initiation																						
M00083 Fatty acid biosynthesis, elongation																						
M00086 beta-Oxidation, acyl-CoA synthesis																						
M00087 beta-Oxidation																						
**Lipid metabolism**																						
M00091 Phosphatidylcholine (PC) biosynthesis, PE => PC																						
M00093 Phosphatidylethanolamine (PE) biosynthesis, PA => PS => PE																						
M00098 Acylglycerol degradation																						
**Purine metabolism**																						
M00048 Inosine monophosphate biosynthesis, PRPP + glutamine => IMP																						
M00049 Adenine ribonucleotide biosynthesis, IMP => ADP,ATP																						
M00050 Guanine ribonucleotide biosynthesis IMP => GDP,GTP																						
**Pyrimidine metabolism**																						
M00052 Pyrimidine ribonucleotide biosynthesis, UMP => UDP/UTP,CDP/CTP																						
M00046 Pyrimidine deg., uracil => beta-alanine, thymine => 3-aminoisobutanoate																						
**Serine and threonine metabolism**																						
M00018 Threonine biosynthesis, aspartate => homoserine => threonine																						
M00020 Serine biosynthesis, glycerate-3P => serine																						
M00555 Betaine biosynthesis, choline => betaine																						
**Cysteine and methionine metabolism**																						
M00017 Methionine biosynthesis, apartate => homoserine => methionine																						
M00021 Cysteine biosynthesis, serine => cysteine																						
M00035 Methionine degradation																						
M00338 Cysteine biosynthesis, homocysteine + serine => cysteine																						
**Branched-chain amino acid metabolism**																						
M00019 Val/isoleucine biosynthesis, pyruvate => val/2-oxobutanoate => isoleucine																						
M00432 Leucine biosynthesis, 2-oxoisovalerate => 2-oxoisocaproate																						
M00535 Isoleucine biosynthesis, pyruvate => 2-oxobutanoate																						
M00570 Isoleucine biosynthesis, threonine => 2-oxobutanoate => isoleucine																						
**Arginine and proline metabolism**																						
M00015 Proline biosynthesis, glutamate => proline																						
M00028 Ornithine biosynthesis, glutamate => ornithine																						
M00844 Arginine biosynthesis, ornithine => arginine																						
**Polyamine biosynthesis**																						
M00134 Polyamine biosynthesis, arginine => ornithine => putrescine																						
M00135 GABA biosynthesis, eukaryotes, putrescine => GABA																						
**Aromatic amino acid metabolism**																						
M00022 Shikimate pathway, phosphoenolpyruvate + erythrose-4P => chorismate																						
M00023 Tryptophan biosynthesis, chorismate => tryptophan																						
**Histidine metabolism**																						
M00026 Histidine biosynthesis, PRPP => histidine																						
M00045 Histidine degradation, histidine => N-formiminoglutamate => glutamate																						
**Lysine metabolism**																						
M00016 Lysine biosynthesis, succinyl-DAP pathway, aspartate => lysine																						
M00527 Lysine biosynthesis, DAP aminotransferase pathway, aspartate => lysine																						
**Other amino acid metabolism**																						
M00027 GABA (gamma-Aminobutyrate) shunt																						
M00118 Glutathione biosynthesis, glutamate => glutathione																						
**Lipopolysaccharide metabolism**																						
M00063 CMP-KDO biosynthesis																						
M00866 KDO2-lipid A biosynthesis, Raetz pathway, non-LpxL-LpxM type																						
**Cofactor and vitamin metabolism**																						
M00115 NAD biosynthesis, aspartate => NAD																						
M00119 Pantothenate biosynthesis, valine/L-aspartate => pantothenate																						
M00120 Coenzyme A biosynthesis, pantothenate => CoA																						
M00121 Heme biosynthesis, plants and bacteria, glutamate => heme																						
M00123 Biotin biosynthesis, pimeloyl-ACP/CoA => biotin																						
M00140 C1-unit interconversion, prokaryotes																						
M00577 Biotin biosynthesis, BioW pathway, pimelate => pimeloyl-CoA => biotin																						
M00846 Siroheme biosynthesis, glutamate => siroheme																						
M00868 Heme biosynthesis, animals and fungi, glycine => heme																						
M00880 Molybdenum cofactor biosynthesis, GTP => molybdenum cofactor																						
**Sulfur metabolism**																						
M00176 Assimilatory sulfate reduction, sulfate => H2S																						
**Nitrogen metabolism**																						
M00531 Assimilatory nitrate reduction, nitrate => ammonia																						
**Polyketide sugar unit biosynthesis**																						
M00793 dTDP-L-rhamnose biosynthesis																						
**Aromatics degradation**																						
M00551 Benzoate deg., benzoate => catechol/methylbenzoate => methylcatechol																						
**Metabolic capacity**																						
M00616 Sulfate-sulfur assimilation																						
M00615 Nitrate assimilation																						

Table S3. Summary of the metagenome-assembled genomes (MAGs) isolated in the present study. Average amino-acid identity (AAI) was calculated by comparing the MAGs with their closest reference genomes identified based on the RAST results. The RefSeq accession number of the reference genomes are provided in parenthesis.

MAG	Completeness (%)	Contamination (%)	GC (%)	N50	Size (bp)	Phyla	Class/Order	Reference genome	AAI (%)
MAG-1640-1.1	80.57	1.536	69.1	8867	5073303	Actinobacteria	Actinomycetales	*Actinosynnema mirum* DSM 43827^T (NC_013093.1)	58.65
MAG-1640-2.1	85.36	3.738	66.8	11294	5751495	Proteobacteria	Rhodospirillales	*Roseomonas gilardii* U14-5 (NZ_CP015583.1)	49.1
MAG-1751-1.1	70.3	3.1	70.8	7414	4752807	Acidobacteria	Bryobacterales	*Candidatus* Koribacter versatilis Ellin345 (NC_008009.1)	40.96
MAG-1957-1.1	85.8	3.48	58.3	67512	4938955	Acidobacteria	Bryobacterales	*Bryobacter aggregatus* MPL3^T (NZ_JNIF01000001.1)	49.77
MAG-1957-2.1	96.2	3.96	53.4	119701	6327585	Chloroflexi	Ktedonobacteria	*Ktedonosporobacter rubrisoli* SCAWS-G2^T (NZ_CP035758.1)	59.01
MAG-1957-3.1	72.22	3.47	51.7	13375	6142618	Chloroflexi	Ktedonobacteria	*Dictyobacter vulcani* W12^T (NZ_BKZW01000001.1)	63.43
MAG-1957-4.1	72.64	0.1	67.2	5165	3007516	Firmicutes	Thermoanaerobacterales	*Ammonifex degensii* KC4^T (NC_013385.1)	41.36
MAG-1957-5.1	98.05	0.9	71.4	79391	5837494	Actinobacteria	Actinomycetales	*Actinosynnema mirum* DSM 43827^T (NC_013093.1)	55.92
MAG-1957-6.1	80.36	1.98	54.8	22264	5685492	Chloroflexi	Ktedonobacteria	*Ktedonosporobacter rubrisoli* SCAWS-G2^T (NZ_CP035758.1)	58.66
MAG-1957-7.1	94.02	1.71	58.9	137749	4659534	Acidobacteria	Bryobacterales	*Candidatus* Koribacter versatilis Ellin345 (NC_008009.1)	47.98
MAG-1957-8.1	70.49	3.3	72.5	10863	4227183	Gemmatimonadetes	Gemmatimonadales	*Gemmatirosa kalamazoonesis* KBS708^T (NZ_CP007128.1)	60.46
MAG-1957-9.1	90.97	4.94	71.3	26163	6509599	Actinobacteria	Actinomycetales	*Streptosporangium roseum* DSM 43021^T (NC_013595.1)	52.26
MAG-1957-10.1	72.61	1.07	71.4	17599	4220629	Actinobacteria	Actinomycetales	*Streptosporangium roseum* DSM 43021^T (NC_013595.1)	52.93
MAG-1957-11.1	89.03	1.72	60.9	88061	3399414	Acidobacteria	Acidobacteriales	*Acidipila rosea* DSM 103428 (NZ_SMGK01000001.1)	54.36
MAG-1957-12.1	73.41	1.72	58.9	22191	4793286	Acidobacteria	Acidobacteriales	*Acidobacterium capsulatum* ATCC 51196^T (NC_012483.1)	57.18
MAG-1957-13.1	95.6	4.09	60.5	13562	5255071	Verrucomicrobia	Verrucomicrobiales	*Prosthecobacter debontii* ATCC 700200^T (NZ_FUYE01000055.1)	44.57
MAG-1957-14.1	96.79	3.19	61.9	29422	6536060	Proteobacteria	Rhodospirillales	*Acidisphaera rubrifaciens* HS-AP3^T (NZ_BANB01001239.1)	61.34
MAG-1957-15.1	94.61	3.54	61.5	71986	3914044	Acidobacteria	Acidobacteriales	*Acidipila rosea* DSM 103428 (NZ_SMGK01000001.1)	57.33
MAG-1957-16.1	99.14	0.86	65.4	109666	3669710	Acidobacteria	Acidobacteriales	*Acidipila rosea* DSM 103428 (NZ_SMGK01000001.1)	57.44

Table S4. Summary of enzymatic functions for CO-, H2-, and formate-oxidation in the Ktedonobacteria reference genomes. Carbon monoxide oxidation: K03518 carbon monoxide dehydrogenase small subunit coxS, K03519 carbon monoxide dehydrogenase medium subunit coxM, K03520 carbon monoxide dehydrogenase large subunit coxL. Formate oxidation: K00122 formate dehydrogenase, K00123 formate dehydrogenase major subunit. Hydrogen oxidation: H_2 dehydrogenase: K00436: NAD-reducing hydrogenase large subunit.

Species	Strain	Isolation source	RefSeq	Collection	Reference	K03518	K03519	K03520	K00122	K00123	K00436
Ktedonobacter racemifer	SOSP1-21^T	European soils	NZ_ADVG00000000.1	DSM 44963	[2]	x	x	x	x	x	x
Dictyobacter aurantiacus	S-27^T	paddy soils	NZ_BIFQ00000000.1	ASM396751v1	[4]	x	x	x	x	x	x
Dictyobacter vulcani	W12^T	volcanic soils	NZ_BKZW00000000.1	NBRC113551T	[3]	x	x	x	x	x	x
Thermosporothrix hazakensis	SK20-1^T	ripe compost	NZ_BIFX00000000.1	ATCC BAA-1881	[5]	x	x	x	x	x	x
Ktedonosporobacter rubrisoli	SCAWS-G2^T	red soils	GCF_004208415.1	DSM:105258	[6]	x	x	x	x	x	x
Tengunoibacter tsumagoiensis	Uno3^T	soil-like granular mass	NZ_BIFR00000000.1	ASM396753v1	[7]	x	x	x	x	x	x
Dictyobacter kobayashii	Uno11^T	soil-like granular mass	NZ_BIFS00000000.1;	ASM396755v1	[7]	x	x	x	x	x	x
Dictyobacter alpinus	Uno16^T	soil-like granular mass	NZ_BIFT00000000.1	ASM396757v1	[7]	x	x	x	x	x	x
Thermogemmatispora aurantia	A1-2^T	geothermal soil	NZ_BKZV00000000.1	ASM897428v1	[8]	x	x	x	x	x	x
Thermogemmatispora onikobensis	NBRC 111776	geothermal soil	NZ_BDGT00000000.1	ASM174828v1	unpublished	x	x	x	x	x	x
Thermogemmatispora carboxidivorans	PM5^T	biofilm	NZ_JNIM00000000.1;	DSM45816T	[1]	x	x	x	x	x	x

Supplementary references

1. King, C.E.; King, G.M. Description of *Thermogemmatispora carboxidivorans* sp. Nov., a carbon-monoxide-oxidizing member of the class Ktedonobacteria isolated from a geothermally heated biofilm, and analysis of carbon monoxide oxidation by members of the class Ktedonobacteria. *Int. J. Syst. Evol. Microbiol*. **2014**, *64*, 1244-1251.
2. Chang, Y.J.; Land, M.; Hauser, L.; Chertkov, O.; Del Rio T.G.; Nolan, M.; et al. Non-contiguous finished genome sequence and contextual data of the filamentous soil bacterium *Ktedonobacter racemifer* type strain (SOSP1-21). *Stand. Genomic. Sci.* **2011**, *5*, 97-111.
3. Zheng, Y.; Wang, C.M.; Sakai, Y.; Abe, K.; Yokota, A.; Yabe, S. *Dictyobacter vulcani* sp. nov., belonging to the class Ktedonobacteria, isolated from soil of the Mt Zao volcano. *Int. J. Syst. Evol. Microbiol.* **2020**, *70*, 1805-1813.
4. Yabe, S.; Sakai, Y.; Abe, K.; Yokota, A.; Také, A.; Matsumoto, A.; et al. *Dictyobacter aurantiacus* gen. nov., sp. nov., a member of the family Ktedonobacteraceae, isolated from soil, and emended description of the genus *Thermosporothrix*. *Int. J. Syst. Evol. Microbiol.* **2017**, *67*, 2615-2621.
5. Yabe, S.; Aiba, Y.; Sakai, Y.; Hazaka, M.; Yokota, A. *Thermosporothrix hazakensis* gen. nov., sp. nov., isolated from compost and description of Thermosporotrichaceae fam. nov. within the class Ktedonobacteria. *Int. J. Syst. Evol. Microbiol*. **2010**, *60*, 1794-1801.
6. Yan, B.; Guo, X.; Liu, M.; Huang, Y. *Ktedonosporobacter rubrisoli* gen. nov., sp. nov., a novel representative of the class Ktedonobacteria, isolated from red soil, and proposal of Ktedonosporobacteraceae fam. nov. *Int. J. Syst. Evol. Microbiol.* **2020**, *70*, 1015-1025.
7. Wang, C.M.; Zheng, Y.; Sakai, Y.; Toyoda, A. Minakuchi, Y.; Abe, K.; et al. *Tengunoibacter tsumagoiensis* gen. nov., sp. nov., *Dictyobacter kobayashii* sp. nov., *Dictyobacter alpinus* sp. nov., and description of Dictyobacteraceae fam. nov. within the order Ktedonobacterales isolated from Tengu-no-mugimeshi, a soil-like granular mass of microorganisms, and emended descriptions of the genera *Ktedonobacter* and *Dictyobacter*. *Int. J. Syst. Evol. Microbi*ol. **2019**, *69*, 1910-1918.
8. Zheng, Y.; Wang, C.M.; Sakai, Y.; Abe, K.; Yokota, A.; et al. *Thermogemmatispora aurantia* sp. nov. and *Thermogemmatispora argillosa* sp. nov., within the class Ktedonobacteria, and emended description of the genus *Thermogemmatispora*. *Int. J. Syst. Evol. Microbiol*. **2019**, *69*, 1744-1750.
